# High School Student Burnout: Is Empathy a Protective or Risk Factor?

**DOI:** 10.3389/fpsyg.2020.00897

**Published:** 2020-05-13

**Authors:** Eleonora Farina, Veronica Ornaghi, Alessandro Pepe, Caterina Fiorilli, Ilaria Grazzani

**Affiliations:** ^1^Department of Human Sciences for Education “R. Massa”, University of Milano-Bicocca, Milan, Italy; ^2^Department of Human Studies - Communication, Education, and Psychology, LUMSA University, Rome, Italy

**Keywords:** student burnout, empathy, satisfaction with school relationships, high school, adolescents

## Abstract

Students’ school burnout has been extensively investigated in relation to interpersonal factors such as peer relations and social adjustment. However, few studies have examined the role of individual traits such as empathic skills. Our aim in this study was to test, within a single comprehensive model, how students’ empathic skills affect their levels of school burnout, both directly and indirectly via satisfaction with school relationships. A sample of 998 high school students (aged 14 to 19 years) took part in this cross-sectional study. Participants completed quantitative self-report measures of school burnout, empathic skills (both cognitive and affective), and satisfaction with school relationships (peers and teachers). Using structural equation modeling, we tested a conceptual model in which emphatic skills were hierarchically associated with satisfaction about school relationships and school burnout, while also controlling for age. The structural equation model offered an excellent fit for the empirical data. Analysis of the total, direct, and indirect effects showed that empathic skills were associated with both satisfaction about school relationships and school burnout. Satisfaction with school relationships appeared to mediate the relationship between empathy and school burnout. Students’ age was also found to have statistically significant effects. The negative effect of high school students’ empathic skills on their risk of school burnout may be prevented or at least reduced by helping them to develop positive and satisfying relationships with both teachers and peers.

## Introduction

A large body of international research has documented the difficulty experienced by high school students in dealing with achievement pressure (Dupéré et al., 2015; Prabhu, 2015). Feeling unable to overcome academic difficulties or manage demanding school events can easily lead adolescents to develop burnout. The concept of burnout was originally formulated to describe reactions to stress in the workplace and, over time, it has been studied across a range of occupational fields. Applying the construct to educational settings suggests that, like work, school requires individuals to engage with multiple achievement pressures. Thus, school burnout may be defined as a response to school-related stress, which becomes chronic when students stably perceive a discrepancy between their individual resources and their personal expectations of success (Parker and Salmela-Aro, 2011; Salmela-Aro et al., 2017). More specifically, burnout levels are due to a cumulative process which may increase or decrease in relation to personal and external support resources.

Since burnout generally emerges in a period of youth that is particularly sensitive to the onset of depressive symptoms, this has led scholars to consider depression and burnout as two overlapping dimensions co-occurring in students’ negative school outcomes. Indeed, longitudinal as well as cross-sectional studies have supported the role of students’ school burnout in predicting their later depressive symptoms development (Salmela-Aro et al., 2009a, 2017). The construct of burnout comprises three dimensions: emotional exhaustion, cynicism about school (which manifests as a generally detached attitude toward, and loss of interest in, one’s studies), and a sense of inadequacy as a student (Salmela-Aro et al., 2009a).

The consequences of school burnout can be serious and noticeable in both the short and the long term. Specifically, burnout has been linked to tedium, poor quality of school life, an external locus of control, self-handicapping, failure-avoidance strategies, depressive-anxious symptoms, low self-esteem, general school maladjustment, a higher risk of dropout, risky behaviors, like gambling, and underachievement (Fimian and Cross, 1986; Covington, 2000; Salmela-Aro and Upadyaya, 2014; Räsänen et al., 2015; Fiorilli et al., 2017). Longitudinal and cross-sectional research has shown that school burnout increases with age, and also that experiencing school burnout during adolescence increases the risk of developing depressive symptoms later in adulthood (Salmela-Aro et al., 2009b; Fiorilli et al., 2017). Thus, in recent years, a mounting body of literature has examined the key factors that can lead to, or protect from, burnout syndrome in high school students. Salmela-Aro et al. (2008) found that negative school climate is positively associated with school burnout, whereas support and motivation offered by teachers can protect students from burnout. A study by Slivar (2001) with 1,868 high school students identified poor family relationships and emotionally-oriented coping as leading risk factors for school burnout. School engagement, on the other hand, can play a positive role in mediating the effects of burnout on academic achievement (Fiorilli et al., 2017). Still other studies have identified a range of protective factors such as problem resolution coping strategies (Yusoff, 2010), high-achieving peer groups (Kiuru et al., 2008), the pursuit of achievement-related goals (Vasalampi et al., 2009), teachers’ autonomy support (Shih, 2015) and social support (Kim et al., 2018; Wang et al., 2018). However, most of the risk factors for school burnout discussed in the literature are school-related interpersonal variables, such as school pressure, peer groups, school engagement, and so on (Walburg, 2014). In contrast, internal factors such as individual traits or attitudes have not yet been comprehensively explored in terms of their possible impact on susceptibility to school burnout, whether directly or indirectly via their interaction with environmental factors. To our knowledge, studies in this area mainly focused on emotional intelligence as key to both the effective management of stressful school situations and school well-being (Davis and Humphrey, 2014; Gugliandolo et al., 2015).

Among the many other individual factors that may be implicated in burnout, empathy is likely to play a crucial role. The concept of empathy has a long and broad history in scientific literature: developmental psychology studies agree in identifying evolutionary trajectories of empathic skills, also related to gender specificities. Concerning this aspect, various scholars support the hypothesis that socialization practices induce girls to enact caring behaviors, whereas boys to perform masculine-typed behaviors, like instrumentality and competitiveness (Leaper, 2015); such differences in empathic skills seem to increase along adolescence (Van der Graaff et al., 2014).

Concerning the focus of the present study, empathy is reported to foster positive relationships, social adjustment and, indirectly, personal well-being (Batson et al., 2007; Eisenberg and Eggum, 2009). However, studies that have examined empathy in relation to burnout have yielded more contradictory findings. Most existing studies on the topic of empathy and burnout have concerned healthcare practitioners, and, although some of them found a direct association between high levels of empathy and a low risk of professional burnout (Gleichgerrcht and Decety, 2013; Yuguero et al., 2017), recent research has begun to reveal a more complex picture. First, it is important to note that empathy is a multidimensional construct: precursors of this approach are the models of Feshbach (1975) and Hoffman (1984), in which the vicarious affective reaction to others’ emotions is mediated by cognitive factors. Davis (1980) proposed an integrated model highlighting the interconnection between cognitive and affective aspects with four constructs. Two of them refer to the individual emotional reaction, which can be oriented toward sharing the emotions of others (empathic concern) or toward understanding one’s own anxiety or concern (personal distress). The other two, more cognitive, refer to decentralization (perspective taking) and imagining oneself in fictitious situations (fantasy). In line with this approach, recent advances in cognitive neuroscience have identified four key components of empathy: affective response, self-other awareness, perspective taking, and emotion regulation (Gerdes et al., 2011). Affective response is a physiological process of affect sharing, or the automatic mirroring of another’s emotional behaviors (Decety and Meyer, 2008). The other three components are cognitive processes that entail: recognizing the difference between another’s distress and one’s own physiological reactions (self–other awareness), using those feelings to engage in deep understanding (perspective taking), and simultaneously regulating one’s own emotions so as not to be overwhelmed (emotion regulation) (Preston and De Waal, 2002). Studies linking empathy and professional well-being vs. burnout found that the cognitive components of empathy – particularly self-other awareness – strongly predict lower levels of burnout. In contrast, other authors reported that an unregulated affective response had the effect of increasing distress in social workers confronted with the suffering of others (Wagaman et al., 2015). An approach aiming at balancing compassion and emotional distance in helping professions seems to prevent burnout: studies on this issue often refer to the concept of detached concern (Lampert and Glaser, 2018), which integrates the empathic concern and the necessary detachment toward patients. The recent longitudinal study by Lampert et al. (2019) on healthcare, teaching and social professionals, confirms that a highly balanced detached concern is the healthiest approach in terms burnout levels.

These findings may also be read in light of Bloom’s recent critical work on empathy (Bloom, 2017). Bloom proposed that affective empathy, or the vicarious experience of others’ feelings, may lead to burnout and exhaustion. He based this claim on experiments in which people were trained in either empathy (trying to feel what others feel) or compassion (trying to develop warm and positive thoughts about others’ distress). Individuals who had received training in empathy displayed higher levels of distress (a risk factor for burnout and avoidant strategies), whereas those who had received training in compassion displayed greater positive affect and resilience (which are linked to pro-sociality and effective coping amidst stress).

It is useful to bear these findings in mind when investigating the potential outcomes of empathic attitudes in students facing stressful school situations. The question that remains to be answered is: does empathy protect students from, or expose them to the risk of, school burnout?

Plausibly, empathy may lead to either exhaustion or a positive attitude toward difficulties, depending on how individuals balance the affective and cognitive components of their empathic competence in specific social contexts (Bloom, 2017). Empathic competence may foster social adjustment, thereby assisting the individual in developing a network of supportive relationships, which – in turn – can help to prevent school burnout (Kim et al., 2018; Wang et al., 2018). The investigation of such relationships starting from adolescence onward could be crucial to better comprehend the origin of these relationships, especially in students who choose school curricula intended to prepare for teaching, psychological or social work. Thus, the main aim of the present study was to investigate, within a single comprehensive model, the direct and indirect effects of affective and cognitive empathy on the risk of school burnout in high school students, while also assessing the potential role of students’ satisfaction with their school relationships and controlling for any effects of student age. We hypothesized that we would find: (1) a direct statistical positive effect of empathy on students’ school burnout (H1), given that high levels of empathy – and particularly its affective component – can lead to a greater risk of burnout (Wagaman et al., 2015); (2) a direct statistical positive effect of empathy on satisfaction with school relationships (H2) (Batson et al., 2007; Eisenberg and Eggum, 2009); and (3) a direct statistical negative effect of satisfaction with school relationships on students’ levels of burnout (H3) (Kim et al., 2018; Wang et al., 2018).

## Materials and Methods

### Participants

The sample comprised 998 (883 girls) students. Participants ranged in age from 14 to 19 years (*M* = 16.30; *SD* = 1.56) and were distributed across the school years as follows: 1st year: *N* = 232; 2nd year: *N* = 235; 3rd year: *N* = 166; 4th year: *N* = 216; 5th year: *N* = 149. Participants were recruited at three high schools (Human Sciences Lyceum) of middle socio-economic status in urban areas in the North of Italy. Human Sciences Lyceum offers a preparation mainly focused on social sciences and humanities. Besides core disciplines like mathematics, science, literature etc., high importance is given to pedagogical and psychological issues. After the diploma, the majority of the students chose university courses to become teachers or social workers. All the contacted schools chose to participate in the study based on their interest in the research project. Written parental consent was obtained for the underage students.

### Instruments and Procedure

Students completed three self-report instruments measuring their burnout, empathic skills, and satisfaction with social relationships at school, respectively. All instruments were in written format and were anonymously administered in the classroom in counterbalanced order. The testing session lasted approximately 15 min.

#### Student’s School Burnout

The School Burnout Inventory (SBI) was developed to evaluate school-burnout in 8th–12th-grade students (Salmela-Aro et al., 2009b). It comprises 9 items, which the student is asked to rate on a 6-point Likert scale (from 1 = completely disagree to 6 = strongly agree). The inventory assesses students’ school-burnout across three different dimensions: exhaustion at school (4 items; range 4–24; i.e., “I feel overwhelmed by my schoolwork”), cynicism about the meaning of school (3 items; range: 3–18; i.e., “I feel that I am losing interest in my schoolwork,” and sense of inadequacy at school (2 items; range: 2–12; i.e., “I often have feelings of inadequacy in my schoolwork”). Participants were administered the Italian version of the instrument which has also been confirmed to have a three-factor structure (Fiorilli et al., 2014). Each student was assigned a total score and a subscore for each of the three dimensions (Cronbach’s alpha > 0.80).

#### Students’ Empathic Skills

Two sub-scales of the Interpersonal Reactivity Index (IRI, Davis, 1983) were administered: Perspective Taking (PT; the tendency to spontaneously adopt the psychological point of view of others; e.g., “I sometimes try to understand my friends better by imagining how things look from their perspective”) and Empathic Concern (EC; tendency to feel sympathy and compassion for unfortunate others; e.g., “I often have tender, concerned feelings”). These sub-scales proved to be the most interconnected dimension of the IRI (Davis, 1983; Albiero et al., 2006), moreover, for the purposes of this study, we found it more interesting to focus on the “other-oriented” aspects of affective and cognitive components of empathy, detected, respectively, by Empathic Concern and Perspective Taking. Participants were asked to express the frequency with which they engaged in the described behaviors, using a 5-point Likert scale (from 1 = Never, to 5 = Always). Cronbach’s alpha was > 0.80 for both scales.

#### Satisfaction With School Relations

Participants were asked to express their level of satisfaction concerning their relations with peers and teachers using a 4-point Likert scale (1 = unsatisfied; 4 = fully satisfied).

### Data Analysis and Conceptual Model

**FIGURE 1:**
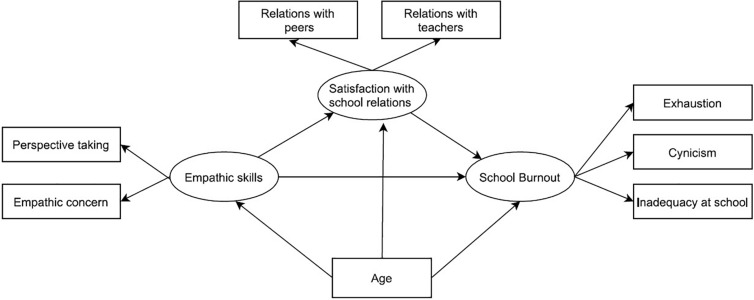
Hypothesized conceptual model of association among empathic skills, satisfaction with school relations and school burnout.

To identify patterns of association between the participants’ empathic skills, satisfaction with school relationships, and school burnout, we chose structural equation modeling (SEM) as our method of analysis. SEM methods are a well-established approach to multivariate data analysis that provide robust estimations of the associations among complex variables (Hair et al., 2014; Ornaghi et al., 2019). In the present study, the proposed conceptual model (see Figure 1) featured three latent endogenous variables: empathic skills, satisfaction with school relationships, and school burnout.

Each variable was operationalized via at least two different empirical indicators as the minimum condition for model identification (Kenny and McCoach, 2003). The total and direct effects of empathic skills on satisfaction with school relationships and burnout were estimated within a single structural model, with age as an external controlling variable. Mahalanobis’ distances (*p* < 0.001) were computed for all variables, with a view to identifying and, if appropriate, removing multivariate outliers. No extreme multivariate values were found. Descriptive statistics and zero-order correlations were calculated. Next, the data were assessed for normality by computing kurtosis and skewness scores. Given that none of the variables under study had indicators falling outside the recommended thresholds of +1 and −1 (see Table 1), the maximum likelihood method was used to estimate the parameters for the structural models (Kline, 2011). With respect to goodness of fit, two classes of indexes (i.e., statistical indicators reflecting the degree of fit between the hypothesized conceptual model and the empirical data) were adopted: absolute fit and relative fit measures. The former included χ^2^ and normed-χ^2^ (NC), where a non-statistically-significant χ^2^ value and NC values of under 2.0 indicate good fit (Hair et al., 2014). The latter comprised the root mean square error of approximation (RMSEA), normed fit index (NFI), non-normed fit index (NNFI), and comparative fit index (CFI). Thresholds for good model fit were: RMSEA < 0.07, NFI > 0.95, NNFI > 0.95, CFI > 0.95 (Hu and Bentler, 1999; Schermelleh-Engel et al., 2003; Marsh and Hau, 2014). To estimate the statistical significance of effects, confidence limits were calculated using both Monte Carlo simulation (MacKinnon et al., 2004) and bootstrapping methods with a set of random samples (*k* = 500).

**TABLE 1:** Main descriptive statistics and zero-order correlations of variables under study.

	**min**	**max**	**m**	**sd**	**Skeweness**	**1**	**2**	**3**	**4**	**5**	**6**	**7**	**8**
(1) Exaustion	4.00	24.00	15.30	4.39	−0.28	–							
(2) Cynicism	3.00	18.00	10.30	3.81	−0.03	0.433**	–						
(3) Inadequacy at school	2.00	12.00	7.15	2.47	−0.20	0.480**	0.687**	–					
(4) Relationship with peers	1.00	4.00	2.99	0.69	−0.39	−0.105**	−0.141**	−0.165**	–				
(5) Relationship with teachers	1.00	4.00	2.70	0.61	−0.82	−0.230**	−0.369**	−0.342**	0.156**	–			
(6) Empathic concern	1.00	5.00	3.90	0.70	−0.65	0.157**	−*0.057*	*0.038*	0.108**	0.114**	–		
(7) Perspective taking	1.00	5.00	3.34	0.78	−0.27	*0.049*	−0.067*	−*0.029*	*0.058*	0.084*	0.294**	–	
(8) Age	14.00	19.00	16.30	1.56	0.16	0.174**	0.221**	0.231**	−0.162**	−0.151**	*0.059*	0.066*	–

*^∗^*p* < 0.01; ^∗∗^*p* < 0.001; non-statistically significant correlations were reported in *italics*.*

## Results

Table 1 shows the main descriptive statistics for the variables under study and the zero-order correlations among them.

The correlational analysis revealed that both perspective taking and empathic concern were positively correlated with satisfaction with school relationships. Regarding the association between empathic skills and school burnout, empathic concern was positively correlated with exhaustion (*r* = 0.16, *p* < 0.001) and perspective taking was negatively correlated with cynicism (*r* = 0.16, *p* < 0.001). Finally, the measures of satisfaction were negatively associated with all dimensions of school burnout, with mean correlations ranging between −0.37 and −0.11. The variable age was correlated (with varying patterns and magnitude of effect sizes) with all the key research variables.

**FIGURE 2:**
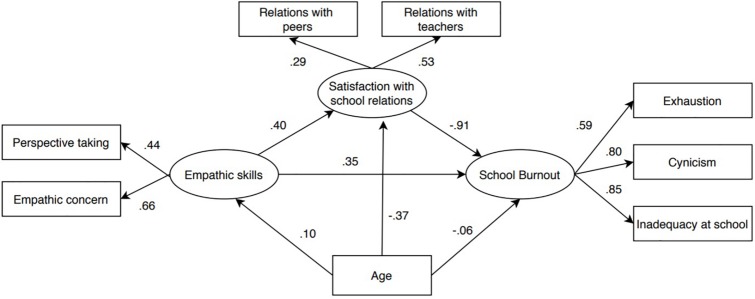
Results of the Structural equation model (SEM). Standardized direct effects were reported.

The SEM analysis (see Figure 2) suggested that the proposed structural model provided an excellent fit for the data: χ^2^(15) = 70.11, RMSEA = 0.059, NNFI = 0.959; CFI = 0.96, SRMR = 0.037. Reading the figure from left to right, we see that empathic skills exerted a direct, positive, medium-sized standardized effect on school burnout (β = 0.35, *p* = 0.003, 95% C.I. [0.789 – 3.92]). This outcome provides support for H1, implying that more empathic students were more likely to experience higher levels of school burnout. Concerning the association between empathic skills and satisfaction with school relations, the standardized direct effect (β = 0.40, *p* = 0.005, 95% C.I. [0.078 – 0.297]) was positive and medium in size. This outcome bore out H2, suggesting that stronger empathic skills were associated with more satisfactory school relationships. Finally (H3), a large, statistically significant, negative direct standardized effect (β = −0.91, *p* = 0.001, 95% C.I. [−18.33 – −7.77]) was found between satisfaction with school relationships and burnout, confirming the role of social relationships (especially with teachers) in mitigating burnout. Interestingly, when the total effect of empathic skills on school burnout was taken into consideration, a more nuanced scenario emerged. The total effect was trivial in size (β = −0.02, *p* = 0.633, 95% C.I. [−0.842 – 0.297]) and not statistically significant. Analysis of the indirect effect of empathy on burnout via satisfaction with relationships (β = −0.02, *p* = 0.005, 95% C.I. [−4.01 – −0.777]) suggests the crucial importance of having satisfactory relationships with others at school. Indeed, the negative indirect effect of empathy via satisfaction with relationships completely compensated for the positive association between empathy and school burnout, indicating a full mediation process. Finally, age was directly and negatively associated with satisfaction about school relationships (β = −0.37, *p* = 0.004, 95% C.I. [−0.063 – −0.029]), with older students reporting poorer satisfaction than younger ones.

## Discussion

In the current study, we investigated the effects of high school students’ empathic skills on their levels of burnout, while also considering the role of satisfaction about school relationships (with both peers and teachers). The main findings were as follows. First, adolescents’ empathic skills and their levels of school burnout were positively correlated; second, satisfaction with school relations was significantly negatively associated with school burnout; third, empathy had an indirect effect on school burnout via satisfaction with school relations; fourth, students’ age also exerted significant effects on the key research variables. We now discuss each of these findings in detail.

The positive association between empathic concern and emotional exhaustion is in line with recent literature that identifies affective empathy as a possible risk factor for burnout (Bloom, 2017). Indeed, high levels of empathic concern may be associated with greater susceptibility to school-related emotional pressures, such as feeling overwhelmed by one’s schoolwork or unable to cope with the multiple demands of school. On the other hand, the negative association between cognitive perspective taking and cynicism is consistent with the notion that the cognitive dimension of empathy protects individuals from stress (Wagaman et al., 2015), whereas cynicism – which may be viewed as a cognitive defense strategy of avoidance/devaluation in the face of stressful events – can prove costly and ineffective over the long term.

The negative link between satisfaction with school relationships and burnout is in line with both the research hypothesis and the existing literature. Students who are more satisfied with the quality of their school relationships are less at risk of burnout, which indirectly confirms that it is important for students to be able to draw support from within the school setting itself (Kim et al., 2018; Wang et al., 2018).

In relation to the main research purpose – that is to say, identifying overall patterns of association between empathic skills, satisfaction with school relationships, and school burnout – the SEM analysis indicated that empathy, especially its affective component, exerted direct positive effects on the participants’ levels of burnout. Affective empathic involvement may therefore represent a risk factor for the development of school burnout, confirming the findings of studies on the helping professions (Wagaman et al., 2015). Empathy, on the other hand, also had positive effects on students’ satisfaction with their school relationships, confirming the value of empathic attitudes for building and maintaining positive and satisfying relationships with others (Batson et al., 2007; Eisenberg and Eggum, 2009). However, satisfaction with relationships was the factor that exerted the greatest effect on burnout: thus, high school students’ satisfaction with their peer relations, but especially with their relationships with teachers, plays a key role in protecting them from the risk of developing burnout. This outcome is in keeping with the literature documenting the key role of peers as a source of support in stressful school situations but shows that positive relationships between teachers and pupils are an even more crucial form of support when it comes to preventing school burnout (Salmela-Aro et al., 2008; García-Moya et al., 2019).

Furthermore, the finding that empathy indirectly impacts the risk of school burnout via satisfaction with relationships suggests that good social relationships at school have the power to compensate for the negative effects of excessive empathic involvement – especially at the affective level - in demanding and potentially stressful situations. This outcome suggests that although high levels of emotional empathy can increase the risk of school burnout in adolescence, this risk will be reduced if students are satisfied with their social relationships at school. Perceiving that they have a good social network at school which they can rely on at times of peak stress, reassures students and helps them to meet the demands of school and cope more effectively in critical situations.

Finally, concerning the effects of age, burnout was found to increase over the high school years, while satisfaction with school relationships declined. This is consistent with reports in the literature that older secondary students are at greater risk of burnout (Fiorilli et al., 2017, 2019), being also less satisfied with the quality of their school relationships.

### Limitations and Future Directions

This study is not without its limitations. First, the number of boys and girls was not balanced, which meant that neither sex, nor gender could be included in the tested model. For this reason, the present results are not generalizable to the population of Italian high school students, but they can be considered as representative of the particular group of adolescents attending Human Science Lyceum. This kind of school mainly prepares for helping professions – at the top for burnout risk – and it is mostly chosen by girls, with a percentage of females (89.5% for school year 2018–2019 in Italy) which is consistent with the one in this study. Despite this specification, future research should consider both to improve sex balance and to detect data from different types of high schools for a more complex understanding of the phenomenon. Second, the results suggested that satisfaction with school relationships mediates the association between empathic skills and school burnout. However, the study’s cross-sectional design precludes us from interpreting the outcomes in terms of cause-effect relations among variables. Future studies should adopt longitudinal and cross-lagged research designs with a view to monitoring the associations among variables across different developmental stages. Third, all data were gathered via quantitative self-report questionnaires and therefore the results may be affected by common-method error variance (CMV; variance attributable to the measurement method rather than to the constructs themselves, Podsakoff et al., 2003). Sources of CMV biases (e.g., response bias or sampling errors) are likely to have been present and, consequently, may have contributed to the strong associations found among the constructs. From this point of view, future research should draw on a greater variety of data sources (e.g., qualitative interviews, on-field observations), adopting a mixed-method approach to further refine our understanding of the dynamics among the constructs under study.

## Conclusion

This study is a small contribution to the investigation of the complex construct of empathy and its potential impact on promoting well-being in adolescence. It would undoubtedly be interesting to broaden the viewpoint, also in consideration of the interest that other perspectives have shown toward this construct, see for example the recent neuropsychological studies about the effects of sleep and its deprivation on empathy in adolescence and young adults (Guadagni et al., 2014, 2018).

Consistently with the hypotheses, we found high school students’ empathic attitudes to exert both direct and indirect effects on their levels of burnout. Empathy – despite its crucial role in creating and maintaining positive relationships and social adjustment – can also have negative effects on personal approaches to coping with stressful and demanding situations. High emotional involvement can lead to the development of typical burnout symptoms, such as emotional exhaustion, cynicism, and inadequacy. Therefore, it appears likely that achieving a good balance between affective and cognitive empathy will enhance the quality of students’ social relationships, which in turn can act as protective factors against burnout.

These findings point up the key importance of fostering positive relationships at high school, especially between students and teachers, with a view to protecting students from burnout. Particularly, it would we worth focusing on specific qualities of such relationships, in order to promote student–teacher connectedness (García-Moya et al., 2019). Furthermore, the results clearly suggest the need for intervention in schools to enhance students’ empathic skills by targeting the cognitive, and not only the affective, dimensions of empathic competence. Finally, it is crucial that such intervention be offered during the early years of high school, with a view to preventing subsequent dissatisfaction with relationships and difficulty coping with the demands of school, thereby fostering students’ school well-being and mental health (Cavioni et al., 2020).

## Data Availability Statement

The datasets generated for this study are available on request to the corresponding author.

## Ethics Statement

Ethical review and approval was not required for the study on human participants in accordance with the local legislation and institutional requirements. Written informed consent to participate in this study was provided by the participants’ legal guardian/next of kin.

## Author Contributions

EF contributed to designing the study, collecting, interpreting, and discussing the data, and writing the manuscript. VO contributed to designing the study, interpreting, and discussing the data, drafting and revising the manuscript. AP made a key contribution to analyzing and interpreting the data. CF and IG contributed to discussing the findings.

## Conflict of Interest

The authors declare that the research was conducted in the absence of any commercial or financial relationships that could be construed as a potential conflict of interest.
